# BC-predict: mining of signal biomarkers and production of models for early-stage breast cancer subtyping and prognosis

**DOI:** 10.3389/fbinf.2025.1644695

**Published:** 2025-09-18

**Authors:** Sangeetha Muthamilselvan, Natarajan Vaithilingam, Ashok Palaniappan

**Affiliations:** 1 Systems Computational Biology Lab, Department of Bioinformatics, School of Chemical and Biotechnology, SASTRA Deemed University, Thanjavur, India; 2 Lincoln City Hospital, United Lincolnshire Hospitals, National Health Service, Lincoln, United Kingdom

**Keywords:** breast cancer heterogeneity, molecular and histological subtype, metastatic disease, machine learning, stage-specific differential gene expression, biomarker signature discovery, explainable AI, integrative multi-omics

## Abstract

**Introduction:**

Disease heterogeneity is the hallmark of breast cancer, which is the most common female malignancy. With a disturbing increase in mortality and disease burden, there remains a need for effective early-stage theragnostic and prognostic biomarkers. In this work, we improved on BrcaDx (https://apalania.shinyapps.io/brcadx/) for cancer vs control screening and examined a cluster of adjoining learning problems in breast cancer heterogeneity: (i) identification of metastatic cancers; (ii) molecular subtyping (TNBC, HER2, or luminal); and (iii) histological subtyping (invasive ductal or invasive lobular).

**Methods:**

We analyzed the transcriptomic profiles of breast cancer patients from public-domain databases such as the TCGA using stage-encoded problem-specific statistical models of gene expression and unveiled stage-salient and progression-significant genes. Using a consensus approach, we identified potential machine learning features, and considered six model classes for each learning problem, with hyperparameter optimization on a training dataset and evaluation on a holdout test dataset. A nested approach enabled us to identify the best model class for each learning problem.

**Results:**

External validation of the best models yielded balanced accuracies of 97.42% for cancer vs normal; 88.22% for metastatic v/s non metastatic; 88.79% for ternary molecular subtyping; and ensemble accuracy of 94.23% for histological subtyping. The model for molecular subtyping was validated on a 26-sample TNBC-only out-of-distribution cohort, yielding 25 correct predictions. We performed a late integration of multi-omics datasets by validating the feature space used in each problem with miRNA profiles, methylation profiles, and commercial breast cancer panels.

**Discussion:**

Pending prospective studies, we have translated the models into BC-Predict that forks the best models developed for each problem in a unified interface and provides a complete readout for input instances of expression data, including uncertainty estimates. BC-Predict is freely available for non-commercial purposes at: https://apalania.shinyapps.io/BC-Predict.

## 1 Introduction

Breast cancer is the most common cancer in women, accounting for 32% of all female cancers globally and 28.2% of female cancers in India (Siegel et al., 2024). With about 2.3 million new cases globally in 2020 (11.7% of total), its incidence surpasses that of lung cancer. The statistics paint a grim portrait of burden of disease: 1 in 4 cancer cases and 1 in 6 cancer deaths globally could be attributed to breast cancer, with 88% higher incidence in transitioned countries relative to transitioning countries (Sung et al., 2021). The risk of a person developing breast cancer depends on many factors like sex (women account for >99.5%), age (>80% occur in postmenopausal women), high-risk family history (upto 30% of cases), and genetic factors. The interplay between weak susceptibility alleles and the other risk factors is key to the etiology of the ‘cancer phenotype’ (Cassidy et al., 2015; Hanahan, 2022). Genetic loci with predisposing mutations include: BRCA1/ BRCA2 (autosomal dominant, 50%–85% life time risk) (Risch et al., 2006), TP53 (Li-Fraumeni syndrome, 80%–90% life time risk) (Allain, 2008), CDH1 (60% life time risk and primarily lobular subtype), STK11 (Peutz-Jeghers syndrome, 50% risk), PTEN (Cowden syndrome with 20%–50% risk (Lindor et al., 2008); Lynch syndrome with 25% risk), PALB2 (partner and localiser to BRCA2, age-dependent risk), ATM, BRIP1, CHEK2 (all about 20% risk) and RAD51C/RAD51D (14%–20% risk). The modifiable lifestyle risk factors include physical inactivity especially post-menopausal obesity (100% additional risk), smoking (24% more risk), alcohol (7% risk for every 10g/day), and combined Hormone Replacement therapy (∼20% further risk depending on length of use/stop) (Manyonda et al., 2022). The prevalence of the risk factors varies by country and region. The typical onset of breast cancer is 60–70 years in western countries, but appears to be anticipated at 40–50 years in countries like India (Bhattacharyya et al., 2020). Data maintained at national registries suggest that the urbanization and growth of cities, ‘modernized’ food habits (e.g., high consumption of ultra-processed foods), and lifestyle changes have contributed to the increased incidence of breast cancer in urban areas, whereas betel quid and tobacco chewing habits have significantly contributed to its incidence in rural areas (*P* = 0.003) (Malvia et al., 2017). These cancers tend to be more aggressive with poorer prognosis (higher grade/size, lymphovascular-invasion positive, triple negative, HER2 positive, node positive, and medullary/metaplastic/micro-papillary/pleomorphic sub-types). The frequent presentation of breast cancer in its advanced and less treatable stages in traditional societies could be traced partly to the inadequate social awareness and extant taboos, leading to subpar survival outcomes. Such conditions tend to compound existing gender inequalities, outdated stereotypes, and burden of disease for whole families, and call for remediation of the situation.

Due to the complexity associated with cancers, a composite feature space is necessary to capture the transformation of cells and subsequent disease progression. This may be balanced with the curse of dimensionality that dominates machine learning. AI models based on whole-genome or whole-exome sequencing may be impractical and uninterpretable. McKinney et al. have developed a mammogram-based AI model for breast cancer screening rivalling radiologist readings, paving the way for AI-based decision support systems (McKinney et al., 2020). Convolutional neural network (CNN) models have been developed for identifying breast cancer samples as well as cancer subtyping based on 7091 genes (Mostavi et al., 2020). CUP-AI-DX includes two models: 1D inception CNN model for classifying cancers of unknown primary based on 817 expression features; and (ii) Random Forest model for breast cancer subtyping based on 5925 expression features (Zhao et al., 2020). Breast cancer subtyping models include learning on PAM50 inferred labels (Bastien et al., 2012) via either functional spectra of gene expression profiles (Gao et al., 2019) or deep convolution of RNAseq and CNV profiles (Mohaiminul Islam et al., 2020). Significant strides have been made towards mechanistic understanding and treatment of breast cancer, which has the most number of FDA-approved molecular panels aimed at early-stage actionable information about the disease. These biomarker panels include OncotypeDx based on TAILORx and RxPONDER studies (Zhang et al., 2022), EndoPredict and EndoPredict Plus (Almstedt et al., 2020), MammaPrint (Soliman et al., 2020), Prosigna (based on PAM50 and OPTIMA study) (Baskota et al., 2021), and Breast Cancer Index (Bartlett et al., 2019). Decision aids like PREDICT, Nottingham Prognostic Index (NPI) and Adjuvant Online based on IHC4 (ER/PR/HER2/Ki67) or IHC4+C (including clinical/pathological features like age, tumour size, grade and nodal status) parameters define the level of clinical risk for adjuvant chemotherapy without relying on tumour profiling tests. The translation of AI models into software-as-medical-devices holds promise for bridging health disparities (Muthamilselvan et al., 2023).

The heterogeneity of breast cancer poses formidable challenges, and individual cancer manifestations vary so much that the available biomarker panels retain validity only in limited settings, thereby leaving a large cohort indeterminate (Güler, 2017). Changes in gene expression and mutations modifying protein activities are etiological molecular events driving the cancer phenotype (Brierley et al., 2016). An integrated precision-medicine approach to early detection, effective therapy and favourable prognosis is necessary. Techniques from the field of machine learning could be highly effective in discerning key features in complex datasets, including gene expression datasets, and learning models that map these features to crucial clinical outcomes related to the diagnosis, prognosis, and treatment of cancers (Kourou et al., 2015). Unsupervised learning techniques have been used to identify subtypes in breast cancer based on gene expression (Horr and Buechler, 2021). The molecular subtype of breast cancer could influence the choice of adjuvant therapy (Johnson et al., 2021; Vaidya et al., 2018). Among the histological subtypes, invasive lobular carcinoma is considered indolent and demands a treatment regimen tailored to the prognostic subtype (Fu et al., 2017). Here we have developed a novel framework for identifying the markers of changes in gene expression profiles across the stages and subtypes of breast cancer, enabling means for differential diagnosis and personalized medicine. These candidate features were utilized to create models that address the multiple challenges in breast cancer heterogeneity: (i) cancer or normal screening; (ii) non-metastatic or metastatic discrimination; (iii) molecular subtyping; and (iv) histological subtyping. Together these models could also enable the prognosis of breast cancer (Fitzgibbons et al., 2000; Rakha et al., 2010). The optimal models for each problem required only a handful of features that could be quantified using experimental techniques such as qRT-PCR. All the models were integrated into BC-Predict, a web-based unified interface for harnessing the models. BC-Predict is available for academic research at: https://apalania.shinyapps.io/BC-Predict. All the Supplementary Information for this study are available at: https://doi.org/10.6084/m9.figshare.25282906.

## 2 Materials and methods

### 2.1 Problems related to the characterization of breast cancer heterogeneity

Four problems related to the delineation of individual breast cancers with respect to the expression data of patient samples were considered:

1. Is the patient sample ‘cancer’ or ‘normal’?
2. If cancer: predict ‘non-metastatic’ (stages I, II or III) or ‘metastatic’ (stage-IV cancer).
3. If cancer: predict the molecular subtype of the cancer.
4. If cancer: predict the histological subtype of the cancer.

**FIGURE 1:**
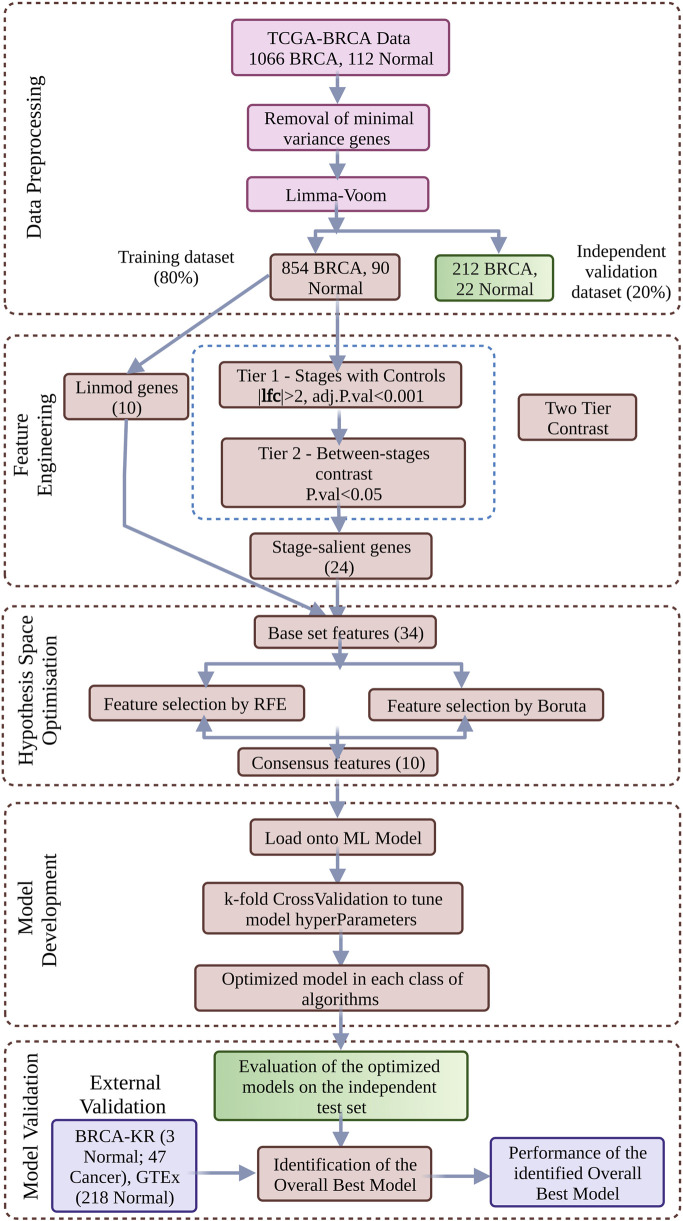
ML model development for Cancer vs. Normal binary classification. Data-driven optimization of a multi-phase workflow, including nested model selection, is shown. Hypothesis space pruning is achieved via feature selection techniques, leading to a consensus gene-signature. Six different classes of machine learning algorithms were considered, with hyperparameter optimization via k-fold cross-validation on the training dataset and model class selection on the holdout test dataset. External validation of the best model yielded a robust assessment of generalizability. Problem-specific substitutions yield workflows adapted to the other problems considered.

A generalized workflow for the problems is depicted in Figure 1.

### 2.2 Dataset preprocessing

Preprocessing was done in a manner similar to Sarathi and Palaniappan (Sarathi and Palaniappan, 2019). The source dataset for all problems modeled here was obtained from the TCGA. Normalised BRCA expression data was acquired using the firebrowse portal (Summary, 2016) (gdac.broadinstitute.org_BRCA.Merge_rnaseqv2__illuminahiseq_rnaseqv2__unc_edu__Level_3__RSEM_genes_normalized__data.Level_3.2016012800.0.0. tar.gz), and RSEM counts were obtained. The patient barcode was matched with the clinical data (gdac.broadinstitute.org_BRCA.Merge_Clinical.Level_1.2016012800.0.0. tar) to extract the patient. stage_event.pathologic_stage variable values that encode the AJCC TNM staging (Giuliano et al., 2018). The sub-stages were then merged to obtain the macro stage categories. Table 1 shows the distribution of sample stages for the breast cancer samples according to the AJCC staging system. It is noted that early-stage BC indicates TNM stage-I or stage-II cancer. Stage-III BC (including T3N1, T4, N2-3) represents loco-regionally advanced BC, whereas T3N0 represents a borderline diagnosis between stages II and III. For the purposes of our study, stages I, II, and III were combined into the ‘non-metastatic’ class.

**TABLE 1:** Stage-wise distribution of TCGA breast cancer samples based on AJCC system, 2018 revision. Numeric suffix is used to indicate the size of tumor (T), number of nodes (N), and presence of metastasis (M).

TCGA stage	TNM classification	Cases	
1	T1N0M0	90	181
1A	T1aN0M0	85	
1B	T1bN0M0	6	
2	T2N0M0	6	616
2A	T2aN0M0	357	
2B	T2b (N0/N1)M0	253	
3	T3N0M0	2	249
3A	T3a (N1/N2)M0	155	
3B	T4(N0/N1/N2)M0	27	
3C	T (any)N3M0	65	
4	T (any)N (any)M1	20	20
Control	—	112	
X		14	
NA	—	8	

The immunohistochemical (IHC) status of oestrogen receptor (ER) and progesterone receptor (PgR), human epidermal growth factor receptor 2 (HER2) oncogene, and Ki-67 (a marker of cell proliferation) are used together to subtype breast tumors into Triple-negative breast cancer (TNBC), HER2-positive, Luminal A and Luminal B (Giuliano et al., 2018; Dai et al., 2015), as shown in Table 2. Where reliable Ki-67 measurements are not available, an alternative assessment of tumor proliferation such as tumor grade could be used to distinguish between ‘Luminal A’ and ‘Luminal B’ (which tends to be HER2 negative). Complete ER, PgR and HER2 IHC metadata were available for 719 samples of the TCGA Breast Cancer dataset, and of these, no sample had information on the Ki-67 labeling index nor on the tumor grade, precluding precise differentiation of luminal subtypes of breast cancers into ‘Luminal A’ or ‘Luminal B’. The luminal subtypes A and B were perforce lumped into one ‘Luminal’ type. The 719 samples were accordingly annotated as 567 ‘Luminal’ (generally Luminal A with Grade 1 or 2 and Luminal B with G3), 115 TNBC (generally Grade 3), and 37 HER2 (generally Grade 3) based on the status of ER, PgR and HER2 extracted from the clinical file (Table 2).

**TABLE 2:** Molecular taxonomy of breast cancer. Luminal A is HER2 negative, whereas Luminal B could be either HER2 positive (accounting for 30% of HER2 positive) or HER2 negative (majority of Luminal B).

S.No.	HER2 status	ER status	PgR status	Ki-67 labelling index	Intrinsic subtype
1	+	+	+	Any	Luminal B (HER2 positive)
		+	–		
		–	+		
2	+	–	–	n/a	HER2+
3	–	+	+	Low (<14%)	Luminal A
				High	Luminal B (HER2 negative)
		+	–	Low (<14%)	Luminal A
				High	Luminal B (HER2 negative)
		–	+	Low (<14%)	Luminal A
				High	Luminal B (HER2 negative)
4	–	–	–	n/a	Triple negative breast cancer (TNBC)

The two most common histological subtypes of breast cancer are infiltrating ductal carcinoma (IDC - no special type) and infiltrating lobular carcinoma (ILC) (Weigelt et al., 2010). ILC tends to be difficult to diagnose, with MR imaging required for determining size and multifocality including contralateral breast (mirror image), and preferential spread to gastrointestinal tract and peritoneum (Winchester et al., 1998). The sample histological subtype is encoded in the clinical metadata ‘patient.histological_type’ with the major values being, ‘infiltrating ductal carcinoma (IDC)’ and ‘infiltrating lobular carcinoma (ILC)’, and minor values including ‘mixed histology’, ‘metaplastic carcinoma’, ‘mucinous carcinoma’, ‘medullary carcinoma’, and ‘other (specify)’.

Genes that had minimal variation in expression across the samples (i.e., σ < 1) were removed. Cancer samples which were missing stage annotation details were removed. The expression dataset was subjected to variance-stabilization using voom function in limma (Law et al., 2014). Linear modeling was then performed. The resulting dataset was split 80:20 into a training set and a holdout testset stratified on the outcome variable of each problem. It is noted that the training dataset for Problem #2 suffered an imbalance in the distribution of the outcome classes (16 metastatic vs. 837 non-metastatic samples), which prompted the application of SMOTE correction (Chawla et al., 2002) (Synthetic Minority Oversampling TEchnique; with arguments: perc. over-represented = 1,000% and perc. under-represented = 300%). Data preprocessing and analysis was done using R (www.r-project.org). The annotated pre-processed final dataset is available as Supplementary File S1.

### 2.3 Construction of feature space

Feature spaces for each problem were constructed using only the training dataset. Initially the differential expression of genes across cancer stages relative to healthy samples was studied using linear modelling with limma (Ritchie et al., 2015):

$$y=\alpha +\beta_{1}X_{1}+\beta_{2}X_{2}+\beta_{3}X_{3}+\beta_{4}X_{4}$$ (1)

Where the independent variables are indicator variables of the sample’s stage, the intercept α is the baseline expression estimated from the controls, and $\beta_{i}$ are the estimated stagewise log fold-change (lfc) coefficients relative to controls.

We then applied a two-level contrast protocol (Muthamilselvan et al., 2023), viz. level-I: stage vs. control and level-II: inter-stages contrast, to produce the following classes of features:

1. Stage-salient genes obtained from all possible pairwise contrasts between the cancer stages using the following model:

$$y=\beta_{0}X_{0}+\beta_{1}X_{1}+\beta_{2}X_{2}+\beta_{3}X_{3}+\beta_{4}X_{4}$$ (2)

Where the controls themselves constitute one of the indicator variables ( $X_{0}$ ), and the $\beta_{i}$ are coefficients estimated from samples of the corresponding annotation only.

1. Monotonically expressed genes obtained from strictly increasing or strictly decreasing mean expression across the cancer stages.

In addition, expression contrasts specific to the problem under consideration were used, namely:

1. contrast of non-metastatic vs. metastatic cancers using the following model modified from Equation 2:

$$y=\mu_{0}X_{0}+\mu_{1}X_{1}+\mu_{2}X_{2}$$ (3)

Where the $\mu_{i}$ are coefficients estimated from samples of the corresponding annotation only.

1. three-way pairwise contrasts between the molecular subtypes; viz. (i) Luminal vs. HER2+, (ii) Luminal vs. TNBC and (iii) HER2+ vs. TNBC using the following model modified from Equation 2:

$$y=\delta_{0}X_{0}+\delta_{1}X_{1}+\delta_{2}X_{2}+\delta_{3}X_{3}$$ (4)

Where the $\delta_{i}$ are coefficients estimated from samples of the corresponding annotation only.

1. contrast of ductal vs. lobular histologies using the following model modified from Equation 2:

$$y=\vartheta_{0}X_{0}+\vartheta_{1}X_{1}+\vartheta_{2}X_{2}$$ (5)

Where the $\vartheta_{i}$ are coefficients estimated from samples of the corresponding annotation only.

The above strategies yielded problem-specific chimeric feature spaces that could span the informative dimensions in each case.

### 2.4 Building problem-specific classification models

A composite feature space comprising the top-ranked genes from the linear model, stage-salient genes, and genes from the problem-specific contrast was subjected to the consensus of two feature selection techniques: (i) Boruta, a wrapper algorithm using Random Forest to select features based on a measure of importance to the outcome variable of interest (Kursa and Rudnicki, 2010); and (ii) Recursive Feature Elimination (RFE), a method that uses backward selection passes to trim the space of predictor variables. The workflow of the machine learning model development in Figure 1 presented in the context of cancer v/s normal was adapted for the non-metastatic v/s metastatic, molecular subtype, and histological subtype classification problems. The training dataset with the final set of features was loaded onto models based on six different algorithms, including Random Forest (ensemble bagging classifier that builds numerous decision trees and ‘bags’ the majority vote), Support Vector Machine (geometric method that finds the maximum margin separating hyperplane in high-dimensional space), k-NN (based on distance-based proximal classes), 1-layer and 2-layer Neural Networks, and XGBoost (ensemble boosting classifier that builds a sequence of classifiers iteratively ‘boosted’ on challenging instances).

### 2.5 Nested model selection

Subsequent to an 80:20 train-test split, algorithm-specific hyperparameter configuration was optimized using 10-fold cross-validation on the training dataset for each of the six algorithms considered. Different algorithm classes were then compared based on their outer-fold testset performance, to identify the optimal algorithm class for each learning problem. The design of such a nested model selection prevents information leakage between model tuning and evaluation, and provides for a more reliable assessment of model generalizability to unseen cohorts than merely cross-validation. Evaluation metrics on the holdout testset as well as external datasets (described below) included balanced accuracy, F1-score, area under ROC (AUROC), Mathews’ correlation coefficient (MCC), and Positive Predictive Value (PPV).

### 2.6 Validation

The overall best model for each problem was validated primarily by performing inference on out-of-domain external datasets. Table 3 shows the datasets used in the development and validation of the ML models for the respective classification problems. In addition, we sought to obtain concurrence for our models from multi-omic signatures, as discussed below.

**TABLE 3:** Datasets used in the modelling of BRCA classification problems. In addition, GSE18549, GSE211167, and METABRIC datasets were also used for external validation in ‘normal vs. cancer’.

S.No	Problem	Dataset used		Sample details	Purpose
1	Normal v/s cancer	TCGA	Training	90 Normal; 854 Cancer	Model building and hyperparameter tuning
			Testing	22 Normal; 212 Cancer	Internal validation
		ICGC (BRCA-KR)		3 Normal; 47 Cancer	External validation
		GTEx		218 Normal	External validation
2	Non-metastatic V/s Metastatic	TCGA	SMOTE- enhanced Training	480 non-metastatic (downsampled from 837); 176 metastatic (upsampled from 16)	Model building and hyperparameter optimization
			Testing	209 non-metastatic; 4 metastatic	Internal validation
		ICGC (BRCA-KR)		47 non-metastatic	External validation
		GSE18549		14 metastatic	External Validation
3	Molecular Subtype	TCGA	Training	454 Luminal; 30 HER2; 92 TNBC	Model building and hyperparameter optimization
			Testing	113 Luminal; 7 HER2; 23 TNBC	Internal validation
		METABRIC		1,415 Luminal; 127 HER2; 299 TNBC	External validation
		GSE211167		26 TNBC	External validation
4	Histological subtype: Ductal v/s Lobular	TCGA	Training	624 Ductal; 162 Lobular	Model building and hyperparameter optimization
			Testing	156 Ductal; 40 Lobular	Internal validation
		The Metastatic Breast Cancer Project		96 Ductal; 19 Lobular	External validation

#### 2.6.1 External validation

##### 2.6.1.1 Normal vs. cancer

We validated model#1 on multiple independent external breast cancer datasets:

- BRCA-KR dataset retrieved from the ICGC DataPortal (https://dcc.icgc.org/) using ‘BRCA’ as the search keyword (Hudson et al., 2010), containing 47 cancer samples and 3 control samples.
- GTEx normal breast dataset (by querying for ‘Breast’ in the “GTEX_phenotype primarysite”) (GTEx Consortium et al., 2013) with 218 control samples.
- GSE18549, GSE211167, and METABRIC datasets.

##### 2.6.1.2 Non-metastatic vs. metastatic

We validated model#2 on two different external breast cancer datasets:

- BRCA-KR dataset described above, with all 47 cancer samples being non-metastatic cancers.
- GSE18549 dataset of metastatic cancers (https://www.ncbi.nlm.nih.gov/geo/query/acc.cgi?acc=GSE18549) (Barrett et al., 2013), with 14 samples having ‘Breast’ as the primary tumor site.

##### 2.6.1.3 Molecular subtyping

We validated model#3 on two different external breast cancer datasets:

- METABRIC a landmark study of breast cancer transcriptomics, available on cBioPortal (https://www.cbioportal.org/study/summary?id=brca_metabric) (Curtis et al., 2012). Breast cancer samples in METABRIC were subtyped as Luminal, HER2, or TNBC based on the IHC status of ER, PgR and HER2 extracted from the METABRIC clinical metadata. This yielded 1,415 Luminal, 127 HER2, and 299 TNBC METABRIC samples. Since METABRIC had used microarray technology to measure gene expression, a platform-specific bias might be induced. To mitigate this bias and obtain data compatible with RNA-Seq technology, we applied the Feature Specific Quantile Normalization (FSQN) technique to the METABRIC data (Franks et al., 2018).
- GEO Dataset GSE211167 (Martini et al., 2022), consisting of only TNBC samples from 26 patients of African ancestry. The dataset was log_2_-transformed prior to serving for model inference.

##### 2.6.1.4 Histological subtyping

We validated model#4 on an external breast cancer dataset from cBioPortal with 96 IDC and 19 ILC samples from the Metastatic Breast Cancer Project (https://www.cbioportal.org/study/summary?id=brca_mbcproject_wagle_2017) (MBCP, 2025).

#### 2.6.2 Late integration of multi-omics data

##### 2.6.2.1 Integration of miRNA analysis

MiRNAs play a crucial role in the regulation of global mRNA expression in both physiological and pathological processes, including the invasion and metastasis of cancer. By exerting control over the expression of target genes, miRNAs act as oncogenes, tumor-suppressive genes, and modulators of distant metastasis in breast cancer. To identify differentially expressed (DE) miRNAs, we used the miRSeq dataset from the same TCGA BRCA cohort (gdac.broadinstitute.org_BRCA.Merge_mirnaseq__illuminahiseq_mirnaseq__bcgsc_ca__Level_3__miR_isoform_expression__data.Level_3.2016012800.0.0.tar.gz). Being a transcriptomics dataset, the miRSeq dataset was treated akin to the mRNASeq dataset, with cancer stage as indicator variable. DE stage-specific miRNAs were revealed upon application of the two-level contrast (stage vs. control level-I contrast and inter-stages level-II contrast). For each identified stage-salient miRNA, the target genes were predicted using multiMiR (Ru et al., 2014), which provides an integration of 14 miRNA-mRNA interaction databases including TargetScan (Ag et al., 2015), miRDB (Wang, 2008), miRanda (Enright et al., 2003), and miRTarBase (Huang et al., 2022). Of the predicted targets for each miRNA, the stage-salient targets were investigated for differential miRNA expression-driven genes.

##### 2.6.2.2 Identification of differential methylation-driven genes (DMDGs)

Epigenetic processes such as methylation could contribute to changes in gene expression and drive pathological processes. To evaluate differentially methylated genes, we used the Level3-processed 450k methylation dataset from the same TCGA BRCA cohort (gdac.broadinstitute.org_BRCA.Merge_methylation__humanmethylation450__jhu_usc_edu__Level_3__within_bioassay_data_set_function__data.aux.2016012800.0.0.tar.gz). The correlation between methylation and expression of the stage-salient genes was analyzed using R MethylMix (Cedoz et al., 2018), with the preset threshold −0.3 and p-value <0.001. Differentially methylated states were identified using significance from Wilcoxon rank-sum testing (adj. p. value <0.05) with an additional effect size filter (>0.1). Genes passing these marker filters were designated as differential methylation-driven genes. Stage-salient differentially methylated genes were identified using the consensus of three stage-informed models, namely Averep, M-value and MethylMix as described (Muthamilselvan et al., 2022).

### 2.7 Development of cascade classifier

**FIGURE 2:**
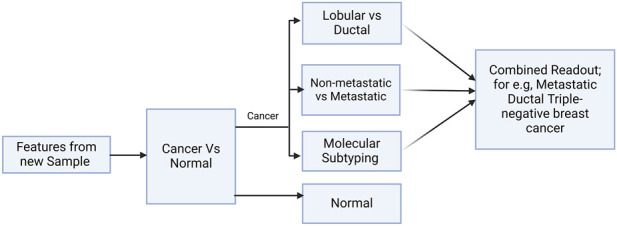
Design of BC-Predict. A schematic of a cascade model for early-stage breast cancer subtyping and prognosis is presented. If the sample is predicted as ‘cancer’ in the first level, it is passed through three more models in the second level that holistically characterize the cancer sample toward personalized medicine.

A prediction pipeline that integrates the predictions from all the models into one combined readout was designed. A schematic for one such cascade model is shown in Figure 2. Based on the decision at the shown fork, the new sample may be taken forward for assessment of metastatic potential and molecular/ histological subtyping. The final readout for a sample from the cascade classifier would consolidate the inference from each model; for e.g., ‘Metastatic triple-negative ductal cancer’. This formed the basis for the development of BC-Predict.

## 3 Results

**FIGURE 3:**
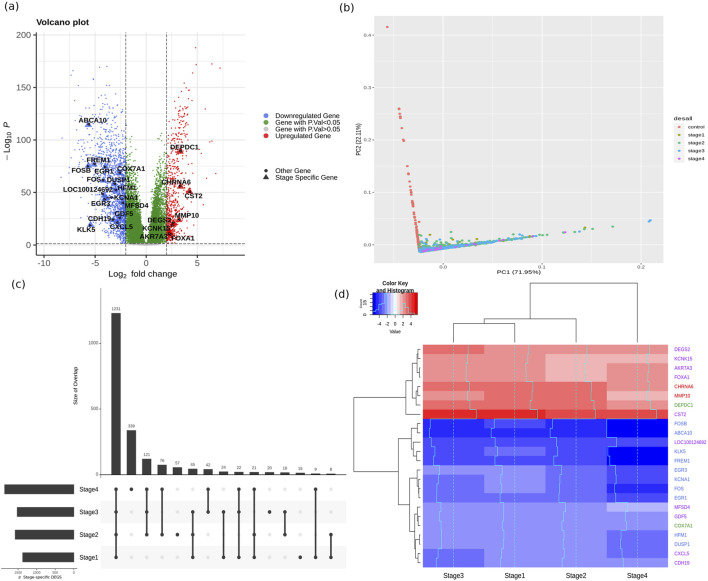
Mining of candidate biomarkers. **(A)** Volcano plot of statistical significance vs log-fold change of differentially expressed genes. Downregulated genes (log-fold change <2) are shown as blue dots, whereas upregulated genes (log-fold change >2) are shown as red dots. Stage-salient genes are highlighted. **(B)** Top two principal components of the expression matrix of the top ten genes from linear modelling. Normal samples can be seen to orient away from cancer samples. **(C)** UpSet plot of the stage-specific contrast analysis illustrating the shared counts of DEGs. **(D)** Heatmap representation of the stagewise expression of the 24 stage-salient genes, with both sample and gene dendrograms. It is seen that the gene dendrogram exhibits two main clusters, corresponding to overexpressed genes (red) and downregulated genes (blue). Euclidean distance metric was used for hierarchical clustering.

**FIGURE 4:**
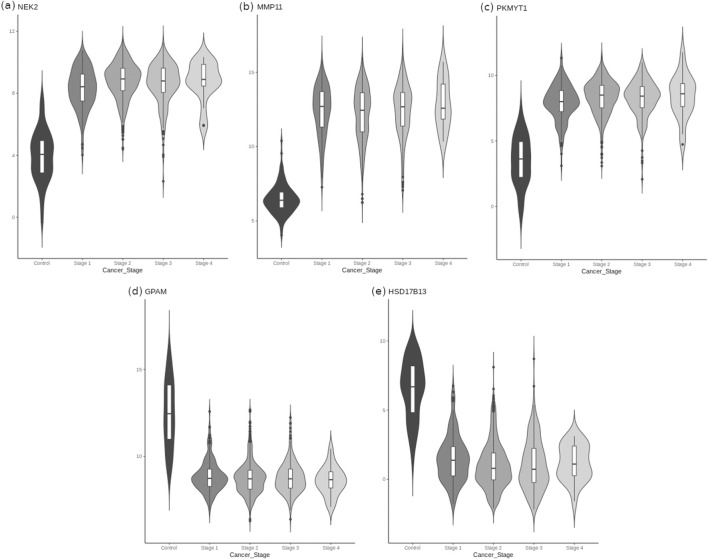
Distribution of expression of the top-ranked genes in linear model sorted by sample stage, to illustrate differential expression patterns. It is seen that **(a)** NEK2 (rank#1), **(b)** MMP11 (rank#2), and **(c)** PKMY11 (rank#3) in the top row are overexpressed in cancers, whereas **(d)** GPAM (rank#4) and **(e)** HSD17B13 (rank#11) in the bottom row are downregulated in cancers. A variability in expression levels of each gene across stages is also seen. The expression violins of all the top 200 genes from the linear model are presented in Supplementary File S3.

The TCGA BRCA dataset consisted of 1,212 samples, each with the measurement of expression of 20532 genes. Post data preprocessing, we obtained an annotated dataset of 1,178 samples x 18880 genes (Supplementary File S1). An adj. p.value cut-off of 0.05 yielded 14838 DE genes in breast cancer samples. Tightening the significance to adj. p-value < 1E-05 still yielded 10167 DE genes, underscoring the persistence of genome instability in the March of cancer (Hanahan, 2022) A volcano plot depicting differentially expressed genes showed significant dispersion (Figure 3a), meaning some genes were much more dysregulated than others. We performed a principal components analysis with the top ten genes from the linear modelling, and found that a clear separation between the normal and cancer samples could be obtained (Figure 3b). This provided some basis for considering top-ranked genes from the linear modeling as candidate cancer-specific features. Table 4 provides information on the top ten genes of the linear modeling, including their regulation status. Information on the top 200 such cancer-specific genes from the linear modelling are provided in Supplementary File S2. Figure 4 shows violin-plot representations of expression distribution of the top ranked genes of the linear model. Violin plots for all the top 200 genes from the linear model are provided in Supplementary File S3.

**TABLE 4:** Top ten genes of the linear model with their stagewise mean log-fold change with respect to control. FDR-corrected significance and inferred regulation type are indicated.

Gene	Stage1 lfc (β_1_)	Stage2 lfc (β_2_)	Stage3 lfc (β_3_)	Stage4 lfc (β_4_)	Adj.P.Val	Regulation status
NEK2	4.34	4.83	4.65	4.82	1.37E-188	Up
MMP11	5.94	5.75	5.96	6.43	3.80E-173	Up
PKMYT1	4.42	4.83	4.73	4.90	1.60E-172	Up
GPAM	−3.57	−3.68	−3.65	−3.85	9.39E-171	Down
CPA1	−4.34	−4.56	−4.28	−4.21	6.39E-170	Down
COL10A1	7.04	6.74	6.95	7.22	3.43E-169	Up
MYOC	−6.06	−6.55	−6.34	−7.17	1.06E-166	Down
KIF4A	4.05	4.54	4.33	4.55	1.61E-164	Up
CA4	−6.63	−7.35	−6.91	−7.11	2.01E-162	Down
LYVE1	−4.76	−5.19	−4.90	−4.91	5.79E-159	Down

Applying the level-I expression filters (|lfc| > 2 and p-value cut-off <0.001) yielded a total of 927 stage-specific genes (74 Stage-I, 238 Stage-II, 90 Stage-III, and 525 Stage-IV specific DEGs, visualized as an Upset plot (Lex et al., 2014) in Figure 3c). For the identification of stage-salient genes two contrasts were applied with stringent criteria and the DEGs identified with different comparisons. This contrast has yielded 2 Stage I salient, 2 Stage II salient, 10 Stage III salient and 20 Stage IV salient genes. Limiting to the top ten stage-IV salient genes (by significance), we finally obtained 24 stage salient genes (Table 5). A heatmap visualization of the stage-salient genes exhibited a systematic differential regulation relative to the controls (Figure 3d). Stage III 4 genes cluster along with Stage I genes and DEPDC1 Stage II with outward CST2. Rest genes from stage III and stage IV form a cluster along with COX7A1 Stage II gene. Violin plots of expression distribution across sample phenotypes for these genes could be found in Supplementary File S4.

**TABLE 5:** Trends in mean expression of stage-salient genes with cancer progression. The inferred regulation status in cancer is noted.

Gene	Stage information	β_0_	β_1_	β_2_	β_3_	β_4_	Adj.P.Val (from contrast)	Adj.P.Val (from control)	Regulation status
CHRNA6	Stage I	−1.67	**3.35**	2.85	2.93	2.21	2.25E-52	7.59E-51	Up
MMP10	Stage I	0.04	**3.19**	2.76	2.61	1.68	5.07E-23	1.66E-24	Up
DEPDC1	Stage II	2.01	2.83	**3.32**	3.03	2.43	3.26E-92	1.39E-89	Up
COX7A1	Stage II	2.36	−2.31	**−2.62**	−2.30	−2.03	3.15E-72	4.39E-69	Down
KCNK15	Stage III	1.99	2.40	1.85	**2.59**	1.72	8.24E-21	5.27E-20	Up
MFSD4	Stage III	1.56	−2.06	−1.96	**−2.32**	−1.79	4.51E-41	2.88E-41	Down
CDH19	Stage III	−3.13	−2.60	−2.58	**−3.19**	−2.61	3.31E-26	1.53E-24	Down
CXCL5	Stage III	−2.03	−2.47	−2.17	**−2.87**	−2.83	5.12E-24	1.30E-22	Down
AKR7A3	Stage III	3.26	2.05	1.52	**2.33**	2.12	1.83E-13	2.55E-12	Up
DEGS2	Stage III	4.82	2.60	2.02	**2.69**	2.27	9.30E-22	1.68E-21	Up
CST2	Stage III	−0.60	4.18	3.57	**4.22**	3.52	2.19E-48	8.75E-52	Up
LOC100124692	Stage III	−2.52	−3.64	−3.60	**−4.13**	−3.83	2.98E-46	8.24E-48	Down
GDF5	Stage III	−1.26	−2.08	−2.31	**−2.63**	−2.24	1.67E-26	3.64E-26	Down
FOXA1	Stage III	7.19	2.09	1.64	**2.32**	1.94	4.81E-13	1.30E-11	Up
EGR3	Stage IV	4.14	−2.33	−2.71	−2.57	**−4.04**	3.53E-18	1.46E-44	Down
FOS	Stage IV	7.27	−2.44	−3.07	−3.09	**−4.19**	3.40E-21	3.50E-62	Down
FOSB	Stage IV	4.71	−3.80	−4.33	−4.30	**−5.66**	9.16E-25	4.51E-76	Down
DUSP1	Stage IV	7.00	−2.13	−2.40	−2.23	**−3.13**	2.51E-19	1.81E-58	Down
FREM1	Stage IV	0.85	−3.67	−4.13	−3.70	**−5.09**	1.29E-23	2.43E-77	Down
EGR1	Stage IV	7.45	−2.72	−3.18	−3.11	**−4.00**	3.63E-23	2.23E-75	Down
HFM1	Stage IV	−3.44	−2.02	−2.24	−2.23	**−3.02**	6.13E-18	1.43E-52	Down
ABCA10	Stage IV	−0.28	−4.38	−4.80	−4.48	**−5.67**	5.63E-33	3.89E-115	Down
KLK5	Stage IV	1.26	−3.21	−3.44	−3.44	**−5.45**	6.93E-20	2.41E-09	Down
KCNA1	Stage IV	−1.69	−2.58	−2.99	−2.81	**−3.93**	3.08E-15	1.99E-45	Down

Bold values indicate coefficients with the largest absolute values, enabling insight into stage-specific expression.

The GO and KEGG pathway analysis was performed for the Stage salient genes to identify over-represented biological processes among these candidate features (complete results in Supplementary File S5; Supplementary File S6, respectively). Genes that were monotonically expressed with cancer progression were identified by observing the trend in mean expression with increasing cancer stage. This yielded 2,246 significantly monotonic genes (1,015 with increasing expression, and 1,231 with decreasing expression). The top 20 such genes with their inferred regulation status are shown in Table 6. A stage-specific gene is said to be contra-regulated when its mean expression is “paradoxical” with cancer progression. There are six patterns of “paradoxical” mean expression, studied in Supplementary File S7. We identified 112 stage-specific genes with such contra-regulation, including one stage-I salient gene (CHRNA6). Contra-regulated genes exhibit unstable expression with cancer progression, and their anomalous behavior might represent possible directions for experimental investigations (Supplementary File S7). Stage-specific DEGs devoid of such contra-regulation suggest a more general role as enhancers of cancer progression.

**TABLE 6:** Top 20 genes with significant monotonic patterns of expression. Intercept, coefficient and adj. p-values from the ordinal model are used. Status indicates monotonic upregulation (UP) or monotonic downregulation (DOWN). The table is sorted by significance (adj.p-value). Adj. R^2^ goodness-of-fit of a stage-ordinal model of expression for each gene is provided.

Gene	Intercept	Coefficient	Adj.P-value	Adj.R^2^	Status
FAM13A	9.842826	−0.62121	1.70E-64	0.2255	Down
GABRD	3.697762	0.889287	2.27E-64	0.2249	Up
KLHL31	6.778289	−0.8667	2.33E-63	0.2217	Down
POC1A	6.587719	0.525973	4.14E-63	0.2209	Up
PAFAH1B3	8.753896	0.602506	1.23E-62	0.2193	Up
SORBS1	11.50753	−0.83632	5.17E-62	0.2174	Down
NIPSNAP3B	6.082268	−0.70387	1.27E-61	0.2161	Down
TMEM220	6.96875	−0.67023	7.56E-60	0.2102	Down
SPTBN1	13.42746	−0.45273	2.81E-59	0.2083	Down
SIK2	10.23114	−0.52331	2.56E-58	0.2051	Down
RECQL4	6.916714	0.743136	1.59E-57	0.2025	Up
C7orf41	10.91012	−0.61324	1.81E-57	0.2023	Down
RAG1AP1	9.736787	0.453142	5.56E-57	0.2001	Up
HSD17B6	4.70826	0.715399	6.98E-57	0.2004	Up
SLC35A2	9.380796	0.311207	7.48E-57	0.2002	Up
CCDC64	6.871398	0.724435	3.72E-56	0.1979	Up
DMD	9.497599	−0.92277	2.47E-55	0.1952	Down
RUSC1	9.565741	0.353172	1.24E-53	0.1897	Up
CXCL2	6.668874	−1.23033	4.45E-53	0.1877	Down
PRR19	4.794229	0.497467	1.87E-52	0.1857	Up

Having completed the mining of signal features, we proceeded to the problem of production of machine learning models. Six model classes were optimized on the train data for each problem and subsequently evaluated on the holdout test to identify the best model class for that problem (Supplementary File S8). A summary of the best overall model for each problem and its validation on the external dataset(s) is presented in Table 7.

**TABLE 7:** The best model class and its performance for each of the problems of interest: (i) normal v/s cancer using ten features, (ii) metastatic v/s non-metastatic using five features, (iii) molecular subtyping using 16 features, and (iv) histological subtyping using 24 features. Nested model selection was used to identify the best model class, with subsequent validation on external datasets. In the case of histological subtype, a voting ensemble of the two models shown was used for the external validation. The RF model for molecular subtyping was externally validated on another 26 TNBC samples, yielding 25 correct predictions. MCC and AUROC values of the best model in each case are scaled to the range [0,100].

S.No	Model	Train	Test	External validation					
		Balanced acc. (%)		Balanced acc. (%)	Specificity	Sensitivity	Precision (PPV)	MCC	AUROC
Normal v/s cancer									
1	NN (1 layer)	99.82	100	97.42	95.74	99.09	95.74	94.84	97.42
Non-metastatic v/s Metastatic									
2	NN (1 layer)	99.17	82.24	88.22	93.87	78.57	91.67	80.87	88.22
Molecular subtype									
3	RF	99.99	91.43	88.79	93.11	84.46	93.63	84.06	90.23
Histological subtype									
4	XGBoost	95.13	88.74	76.92	53.85	100	93.81	71.07	76.92
5	NN (1 layer)	96.97							

### 3.1 Normal v/s cancer

The workflow for this learning problem is shown in Figure 1. Stratified sampling of the TCGA BRCA dataset based on the class ‘cancer’ or ‘normal’ yielded a training dataset of 90 Normal and 854 Cancer samples, and a test dataset of 22 Normal and 212 Cancer samples. The 24 stage-salient genes from the contrasts shown in Equation 2 (namely CHRNA6, MMP10, DEPDC1, COX7A1, KCNK15, MFSD4, CDH19, CXCL5, AKR7A3, DEGS2, CST2, LOC100124692, GDF5, FOXA1, EGR3, FOS, FOSB, DUSP1, FREM1, EGR1, HFM1, ABCA10, KLK5, KCNA1) were combined with the top 10 linear modelling genes from Equation 1 (namely NEK2, MMP11, PKMYT1, GPAM, CPA1, COL10A1, MYOC, KIF4A, CA4, LYVE1) to obtain 34 base features for feature selection. Application of the RFE procedure identified ten features for model development, including two stage-salient genes (FREM1, ABCA10) and eight genes from the linear model (NEK2, MMP11, PKMYT1, GPAM, CPA1, COL10A1, CA4, LYVE1). Of the six ML models trained, four models yielded >99% balanced accuracy on the training set. Subsequent evaluation on holdout testset identified only one model class with 100% accuracy, namely the neural network with one hidden layer model (Supplementary File S8). The model was re-built using the full dataset and validated on external datasets: (i) BRCA-KR, yielding a balanced accuracy ∼94.00%; and (ii) GTEx, yielding ∼100% accuracy (all correct predictions). Together, the model yielded an overall balanced accuracy ∼97.42% on external validation (Table 7). The details could be found in Supplementary File S9, along with the prediction probabilities for all instances in both the external validation. Prediction probability is a measure of the strength of evidence for the predicted class, and based on the distribution of its values, recommendations for evidence of the predicted class may be generated. It was observed that correct predictions were supported by very strong prediction probabilities (>0.9) relative to incorrect predictions.

### 3.2 Non-metastatic v/s metastatic

The workflow for this learning problem is a variation on Figure 1, and available in Supplementary File S10. Stratified sampling of the TCGA BRCA dataset based on the class ‘non-metastatic’ or ‘metastatic’ yielded a training dataset of 837 non-metastatic and 16 Metastatic samples, and a test dataset of 209 non-metastatic and 4 Metastatic samples. SMOTE balancing of the training dataset yielded a dataset with 480 non-metastatic and 176 Metastatic samples. The contrast shown in Equation 3 between non-metastatic and metastatic samples in the SMOTE-balanced dataset produced two lists of genes, one sorted by log-fold change and the other by significance (adj. p-value). The consensus of the top 50 genes from the two lists identified 15 features (namely SRMS, OXT, MMP27, LOC158696, C4orf26, CECR4, ANKRD55, GALNTL6, KRTAP3-1, FAM69C, AFP, CCDC33, SLC5A5, CXorf48, RGS7), to which were added the six top genes by significance missing in the consensus (namely GIP, SSX5, LOC100101938, C9, ASZ1, COX8C). Finally, these 21 genes were pooled with the 24 Stage-salient genes discussed in Cancer V/s Normal classification problem, to obtain 45 base features for feature selection. Application of the Boruta protocol yielded 14 features, while application of RFE procedure yielded just five features. The five RFE features were a subset of the features identified by Boruta, thus we obtained five consensus features for model development, namely DEPDC1, FOSB, DUSP1, MMP27 and ABCA10. Of the six different ML models trained, three models yielded >99% balanced accuracy on the training set. Subsequent evaluation on the holdout testset identified the neural network with one hidden layer model as the best performing model class, with 82.24% balanced accuracy (Supplementary File S8). The model was re-built using the full dataset and validated on the BRCA-KR and GSE18549 datasets, yielding an overall balanced accuracy ∼88.22% on the external validation (Table 7). The details could be found in Supplementary File S10, which includes the prediction probabilities for all instances in the external validation. On inspection of the distribution of prediction probabilities, correct predictions were found to be supported by high values (>0.75) relative to incorrect predictions.

### 3.3 Molecular subtype classification

**FIGURE 5:**
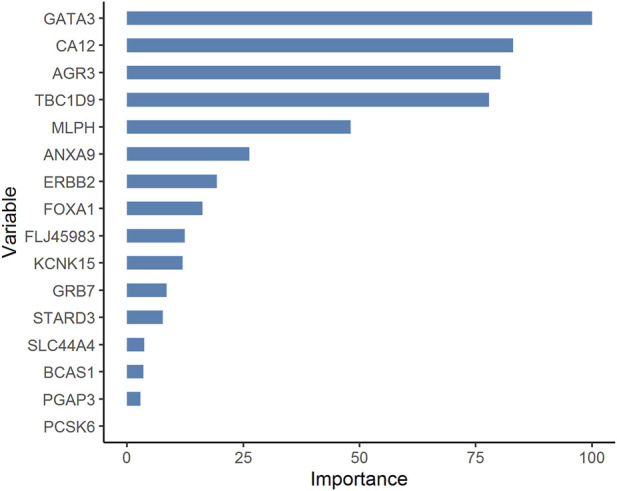
Importance ranking of features used in developing the molecular subtype model. The scores are normalized with respect to the top-scoring feature, GATA3, and presented in the sorted order.

The workflow for this learning problem is a variation on Figure 1, and available in Supplementary File S11. Stratified sampling of the TCGA BRCA dataset based on the molecular subtype class (‘Luminal’ or ‘TNBC’ or ‘HER2’) yielded a training dataset of 434 Luminal, 30 HER2 and 92 TNBC samples, and a test dataset of 113 Luminal, 7 HER2 and 23 TNBC samples. The three-way pairwise contrasts shown in Equation 4 between the molecular subtypes; viz. (i) Luminal vs. HER2, (ii) Luminal vs. TNBC and (iii) HER2 vs. TNBC; yielded subtype-specific genes, from which the top ten genes of each subtype (by significance) were pooled together to obtain 30 base features for feature selection (namely MLPH, AGR3, CA12, TBC1D9, AGR2, TFF3, SIDT1, FZD9, BCAS1, CXorf61, ERBB2, PGAP3, STARD3, C17orf37, GRB7, PSMD3, PCSK6, PNMT, TCAP, LOC150622, GATA3, ANXA9, FLJ45983, PRR15, FOXA1, DEGS2, SLC44A4, ZMYND10, KCNK15, NAT1). Application of the Boruta protocol did not identify any redundant feature, whereas application of RFE procedure yielded 16 features. These 16 features were identified as the consensus features for model development, namely GATA3, AGR3, CA12, TBC1D9, ERBB2, MLPH, KCNK15, ANXA9, FLJ45983, GRB7, PGAP3, STARD3, SLC44A4, PCSK6, FOXA1 and BCAS1. Of the six different ML models trained, the Random forest model provided superlative performance on both the training and outerfold test sets, with balanced accuracies of >99% and 91.43% respectively (Supplementary File S8). The model was re-built using the full dataset and was validated on the METABRIC dataset, yielding a balanced accuracy ∼88.79% (Table 7). Availability of the TNBC-only dataset provided an opportunity to execute a second out-of-cohort validation, yielding correct identification of 25 TNBC samples out of the total 26 samples (96.15% accuracy). The details could be found in the Supplementary File 11, including the prediction probabilities for all instances in the METABRIC and TNBC external validation datasets. On inspection of the distribution of prediction probabilities, correct predictions were found to be supported by high values (>0.7) relative to incorrect predictions. We investigated the 16 features used in the RandomForest model for feature importance based on mean decrease in Gini score in R caret (Kuhn, 2008). The top five features contributing to the model performance were identified as GATA3, CA12, AGR3, TBC1D9, and MLPH (Figure 5).

### 3.4 Histological subtype classification

Stratified sampling of the TCGA BRCA dataset based on the histological subtype (‘IDC’ or ‘ILC’) yielded a training dataset of 624 IDC and 162 ILC samples, and a test dataset of 156 IDC and 40 ILC samples. The contrast shown in Equation 5 between the ductal and lobular histologies was used to detect differentially expressed genes between the two histologies, specifically applying a log-fold change threshold, |lfc| >2, to binarize genes useful as features. This obtained 62 base features for feature selection. Application of the Boruta protocol yielded 58 features, while application of the RFE procedure yielded 24 features. The 24 RFE features were a subset of the Boruta features, thus we obtained 24 consensus features features for model development, namely ADCY5, ALDH1L1, ANKRD43, C1orf64, C7, CAPN8, CCL14, CDH1, CIDEA, CTSG, DARC, F7, FXYD1, HPX, IGFN1, MMP1, PEBP4, PLCXD3, PROL1, SHROOM1, TFAP2B, TFF1, TNNT3, and WNK4. Of the six different ML models trained, four models yielded >95% balanced accuracy on the training set. Subsequent evaluation on the holdout testset identified XGBoost as the best performing model class, with 84.94% balanced accuracy (Supplementary File S8). To mitigate overfitting to the larger IDC class at the expense of the ILC class, we sought to combine the XGBoost model with the 1-layer neural network model, producing a voting ensemble classifier with a slightly better 88.74% balanced accuracy on the holdout testset (Table 7). The ensemble model re-built using the full dataset was validated on the external dataset: brca_mbcproject_wagle_2017, encoding both the histological subtypes of interest (IDC and ILC) as well as other subtypes such as ‘mixed histology’, ‘DCIS’ (ductal carcinoma *in situ*), and ‘NOS’. Predictions were accepted if the two models of the ensemble agreed on the predicted class. If the models disagreed on the predicted class, then the predictions were rejected as ambiguous. Such instances represent challenges to the ensemble classifier whose resolution might not be simple. Omitting the eleven such instances from the external dataset, we obtained correct predictions on all 91 IDC samples as well seven (out of thirteen) ILC samples, yielding an ensemble accuracy ∼94.23% and balanced accuracy ∼76.92% (Table 7). Even with ensembling, generalization errors persisted in learning the ILC class, with an imbalance in the type-II error between the two classes. The details could be found in Supplementary File 12, including the prediction probabilities for all instances in the external validation. On inspection of the distribution of prediction probabilities, correct predictions were found to be supported by high values (>0.7) relative to incorrect predictions. Histological subtyping from molecular features has remained a refractory learning problem, and we have made our models and code freely available for non-commercial use (www.github.com/apalania/BC-Predict_Histological).

### 3.5 Validation with miRNA analysis

Stage-salient miRNA were identified using the two-level contrasts of the miRNA expression data, and then their targets were identified using the R multiMiR library (Supplementary File S13). Based on these results, we determined the concordance between the regulatory miRNAs and their target genes. Temporal concordance in expression exists if the salience in miRNA expression is at least as early as the salience in target gene expression. If the expression pattern of miRNA is discordant with its target gene, a paradoxical aberration with a protective function is possible. Table 8 summarizes the validation of stage-salient gene expression from the angle of miRNA expression. Concordance between the mRNA and miRNA in the direction of expression as well as the temporal dimension is achieved for 13 stage-salient genes: MMP10, DEPDC1, CDH19, FOXA1, DEGS2, CST2, AKR7A3, EGR1, EGR3, FOS, FOSB, FGF2, and HCN2. The key regulatory miRNAs decoded by stage included 25 stage-salient miRNAs (Supplementary File S13), appearing to regulate most of the stage-salient genes. Stage-salient miRNA that were fully concordant with target mRNAs included hsa-miR-182-5p, hsa-miR-210-3p, hsa-miR10b-5p, hsa-miR-200a-5p, hsa-miR-96-5p, hsa-miR-21-5p, hsa-miR-133a-3p, hsa-miR-335-5p, hsa-miR-204-5p, and hsa-miR-145-5p. Further, four of the stage-salient miRNAs regulated genes that featured in the ML models, namely hsa-miR-210-3p, hsa-miR10b-5p, hsa-miR-200a-5p, and hsa-miR-96-5p. Only five stage-salient miRNAs displayed no overlap between their targets and stage-salient genes, and conversely, eleven stage-salient genes were predicted to be free of regulation by a stage-salient miRNA (namely COX7A1, DACT2, KCNK15, MFSD4, DSC3, KLK5, KRT15, LOC100124692, ABCA10, MAPK8IP2, and MASP1). The complete and fully detailed analysis could be found in Supplementary File S13.

**TABLE 8:** Putative target stage-salient genes mapped with their regulatory stage-salient miRNA. Concordance in expression is noted if miRNA overexpression is observed with target gene downregulation or vice-versa. Evaluation of temporal concordance is useful if concordance in expression exists. If there is no concordance in expression, temporal concordance is not evaluated. Genes that display concordance with regulatory miRNA in the direction of expression as well as temporal dimension are emphasized. Target stage-salient genes that represent features used in the ML models are *italicized*. Upregulated miRNAs denote candidate oncomiRs, whereas downregulated miRNAs denote candidate TSmiRs.

S.No	Gene			Regulatory miRNA		
	Name	Expression	Salience	Name	Concordance	
					Expression	Temporal
1	CHRNA6	Up	Stage I	hsa-miR-452-3p	Yes	No
2	**MMP10**	**Up**	**Stage I**	**hsa-miR-182-5p**	**Yes**	**Yes**
				hsa-miR-210-3p	Yes	No
3	***DEPDC1***	***Up***	***Stage II***	***hsa-miR-200b-3p***	Yes	Yes
				***hsa-miR-210-3p***	Yes	Yes
				***hsa-miR10b-5p***	Yes	Yes
				***hsa-miR-200a-5p***	Yes	Yes
				*hsa-miR-96-5p*	No	—
4	**CDH19**	**Down**	**Stage III**	**hsa-miR10b-5p**	No	—
				**hsa-miR-182-5p**	No	—
				hsa-miR-335-5p	No	—
5	GDF5	Down	Stage III	hsa-miR-21-5p	Yes	No
				hsa-miR-335-5p	No	—
				hsa-miR-182-5p	No	—
6	**FOXA1**	**Up**	**Stage III**	**hsa-miR-200a-3p**	Yes	Yes
				hsa-miR-141-3p	No	—
7	**DEGS2**	**Up**	**Stage III**	**hsa-miR-200b-3p**	Yes	Yes
8	**CST2**	**Up**	**Stage III**	**hsa-miR-210-3p**	Yes	Yes
				hsa-miR-335-5p	Yes	No
9	**AKR7A3**	**Up**	**Stage III**	**hsa-miR-210-3p**	Yes	Yes
10	CXCL5	Down	Stage III	hsa-miR10b-5p	No	—
11	**EGR1**	**Down**	**Stage IV**	**hsa-miR-21-5p**	Yes	Yes
				**hsa-miR183-5p**	Yes	Yes
				hsa-miR-204-5p	No	—
				hsa-miR-133a-3p	No	—
				hsa-miR-452-5p	No	—
				hsa-miR-224-5p	No	—
				hsa-miR10b-5p	No	—
				hsa-miR-210-3p	No	—
				hsa-miR-182-5p	No	—
12	**EGR3**	**Down**	**Stage IV**	**hsa-miR183-5p**	Yes	Yes
				hsa-miR-335-5p	No	—
				hsa-miR10b-5p	No	—
				hsa-miR-182-5p	No	—
13	***FOSB***	***Down***	***Stage IV***	***hsa-miR183-5p***	Yes	Yes
				*hsa-miR-224-3p*	No	—
				*hsa-miR-224-5p*	No	—
				*hsa-miR-200b-3p*	No	—
14	KLK7	Down	Stage IV	hsa-miR-335-5p	No	—
				hsa-miR-182-5p	No	—
15	*DUSP1*	*Down*	*Stage IV*	*hsa-miR10b-5p*	No	—
				*hsa-miR-200b-3p*	No	—
				hsa-miR-200b-3p	No	—
17	**FOS**	**Down**	**Stage IV**	**hsa-miR-196a-5p**	Yes	Yes
				**hsa-miR183-5p**	Yes	Yes
				hsa-miR-335-5p	No	—
				hsa-miR10b-5p	No	—
				hsa-miR-139-5p	No	—
				hsa-miR-182-5p	No	—
18	KCNA1	Down	Stage IV	hsa-miR-210-3p	No	—
19	**FGF2**	**Down**	**Stage IV**	**hsa-miR-196a-5p**	Yes	Yes
				**hsa-miR-96-5p**	Yes	Yes
				hsa-miR-145-5p	No	—
				hsa-miR-133a-3p	No	—
				hsa-miR10b-5p	No	—
				hsa-miR-210-3p	No	—
				hsa-miR-182-5p	No	—
20	**HCN2**	**Up**	**Stage IV**	**hsa-miR-133a-3p**	Yes	Yes
21	KIT	Down	Stage IV	hsa-miR-335-5p	No	—
22	*FREM1*	*Down*	*Stage IV*	*hsa-miR-335-5p*	No	—
23	HFM1	Down	Stage IV	hsa-miR-335-5p	No	—

Bold values indicate gene-miRNA combinations with double concordance, in the direction of expression as well as temporal dimension.

### 3.6 Validation with methylation analysis

Aberrant methylation in the core/ proximal promoter regions as well as enhancers could have profound regulatory effects on gene expression. We obtained a total of 22 stage-salient DMGs from the consensus of Averep, Mvalue, and MethylMix procedures: 1 stage-I salient DMG (VOPP1), 8 stage-II salient DMGs (HS3ST3B1, CPLX1, EGR1, GMDS, ITPKB, TGFB1I1, C6orf145, SHC1), 10 stage-III salient DMGs (BTLA, TNFAIP2, PHYHIPL, LYN, MAML2, C16orf62, GPRC5B, CAPN9, AIPL1, AGAP1), and 4 stage-IV salient DMGs (CNP, TSPYL5, SLC7A5, HCN2). Salient methylation of a gene is an epigenetic mechanism to tune gene expression and would precede changes in its expression. In this respect, the stage-II salient methylation of EGR1 possibly set the stage for its stage-IV salience (minimization) in expression. It is observed that the stage-IV salient hypermethylation of HCN2 was at odds with its stage-IV salient overexpression.

**FIGURE 6:**
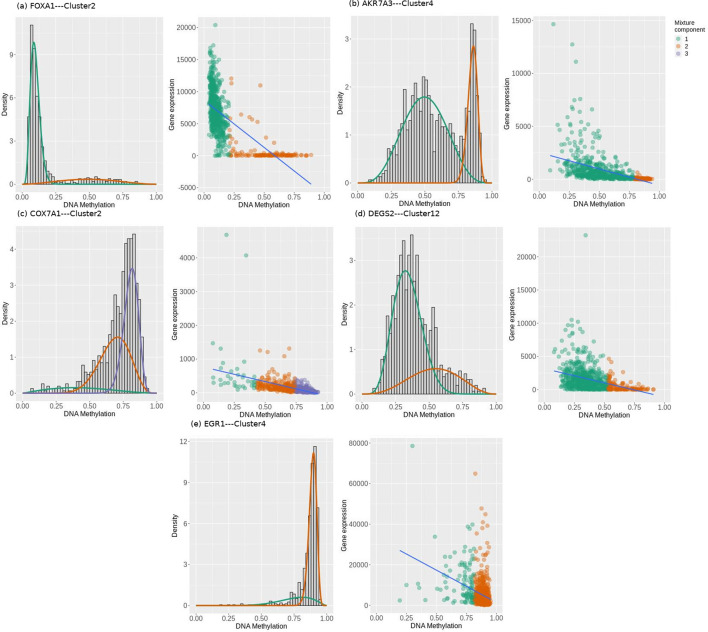
Mixture model of methylation densities, and scatter of expression vs methylation for the respective cluster of each stage-salient differential methylation-driven gene. **(a)** FOXA1 **(b)** AKR7A3 **(c)** COX7A1 **(d)** DEGS2 and **(e)** EGR1. Density plots include mixture components in orange, green, and purple, two for each of FOXA1, AKR7A3, DEGS2, and EGR1, and three for COX7A1. Bayesian Information Criterion was used for estimating the number of mixture components. Scatter plots revealed a consistent negative correlation between DNA methylation and gene expression, marked by different colors for mixture components. Visualized using MethylMix.

Mining the methylation patterns of all stage-salient genes for differential methylation-driven genes revealed five transcriptionally predictive genes negatively correlated with gene expression, namely AKR7A3, COX7A1, DEGS2, EGR1, and FOXA1 (Figure 6). Four of these genes exhibited two-component mixtures of methylation distribution, indicating a probable shift in methylation levels in cancer samples relative to healthy ones. COX7A1 showed three-component mixtures of methylation distribution, indicating a reliance on methylation to achieve regulatory fine-tuning. Table 9 summarizes the methylation patterns for these five genes, showing the correlation size with expression and if the correlation is concordant as well. In the epigenetic context, the methylation pattern of a gene could be deemed concordant with its expression if maximal methylation is observed *ahead of* minimal mRNA expression. FOXA1 mRNA expression is at odds with both its epigenetic profiles (methylation and miRNA), suggesting that epigenetic modulation was being used to restore FOXA1 aberrant expression. Concordance in methylation is observed for AKR7A3, DEGS2, EGR1, and COX7A1, providing strong support for their stage-salience. The above genes except COX7A1 were also concordantly modulated by stage-salient miRNAs. Such findings lead to a belief in the existence of concert between the different layers of omics, adding ‘definiteness’ to gene expression on the path to phenotypic states. Further investigations could shed light on the emergent hypotheses in the future. The mixture decomposition of methylation patterns of the remaining stage-salient genes is provided in Supplementary File S14. It could be seen, for e.g., that the methylation of ABCA10 is positively correlated with its expression, escaping clear interpretation.

**TABLE 9:** Summary of the stage-salient differential methylation-driven genes. Since the methylation of each gene was assayed at a variable number of CpG probe locations, the methylation patterns at different probes for a given gene were clustered based on Pearson’s correlation coefficient cut-off (>0.7). Significant clusters were used to obtain values for: effect size of differential methylation across mixture components, significance of the methylation pattern, coefficient of correlation between expression and methylation, and concordance. Sign of the DM effect signifies the type of aberrant methylation (hyper/ hypo) across the mixture components.

Gene of interest	CpG sites		Significant cluster		DM effect size	p-value	Type of DM	Correlation with expression	Concordance
	Probes	Clusters	ID	Probes					
FOXA1	18	10	Cluster2	5	0.373	1.25E-98	Hyper	−0.66	No
AKR7A3	14	6	Cluster4	1	−0.321	9.89E-48	Hypo	−0.49	Yes
COX7A1	4	2	Cluster2	3	0.413	3.36E-45	Hyper	−0.48	Yes
DEGS2	15	13	Cluster12	1	−0.157	1.56E-25	Hypo	−0.36	Yes
EGR1	13	11	Cluster4	2	0.185	1.21E-23	Hyper	−0.35	Yes

## 4 Discussion

External validation of the models on out-of-domain cohorts suggested that they may be robust to distribution shifts in expression profiles that characterize demographic changes. In a recent study, we applied dimensionality reduction and unsupervised learning to the space of nine expression features (viz. NEK2, PKMYT1, MMP11, CPA1, COL10A1, HSD17B13, CA4, MYOC, LYVE1) and addressed the ‘cancer’ vs. ‘normal’ binary classification, producing BrcaDx (https://apalania.shinyapps.io/BrcaDx) (Muthamilselvan and Palaniappan, 2023) with a balanced accuracy of 95.52% on the BRCA-KR and GTEx. Here we have used a supervised learning approach to the same problem (Figure 2), and derived ten features, including ABCA10, GPAM, FREM1, and the first seven features noted in the prior BrcaDx model. This has yielded a balanced accuracy of 97.42% on the same external datasets, constituting a significant improvement. Beyond the performance improvement, it is noted that BrcaDx suffers from the relative opaqueness of surrogate biomarker spaces (viz. principal components) in its implementation, which tend to obscure interpretation. Other recent advances for discriminating breast cancer from normal samples include a supervised learning model of 20 biomarkers, which was validated on only an internal test set with a balanced accuracy that does not exceed 86% (Taghizadeh et al., 2022). BC-Predict and BrcaDx are both reproducible and interestingly share no common biomarkers with these earlier models.

### 4.1 Literature discussion

We searched Pubmed (www.pubmed.gov) using the keyword: “breast cancer” AND “stage specific” AND “gene”, and found a handful of known stage-specific genes. TIEG (or KLF10) is an anti-metastasis/ tumor-suppressor gene, which inhibits invasive breast cancer by blocking EGFR transcription in the EGFR signalling pathway (W et al., 2012). Stage-specific expression of KLF10 in breast cancer biopsies has been published, with sustained downregulation leading to complete absence of expression in invasive subtypes (Subramaniam et al., 1998). Here KLF10 expression is found to be decreasing with stage relative to the normals. γ-Synuclein (SNCG) expression is strongly correlated with the stages of breast cancer, showing little expression in normal or benign samples and increasing expression with cancer stage, and detectable only in a subset of patients (Wu et al., 2003). Here we find increasing expression of SNCG in late-stage cancers, but downregulated with respect to expression in normal samples, which is a contrarian finding.

#### 4.1.1 Top genes from linear models

Players in cell cycle regulation featured among the top genes of the linear model, namely NEK2, PKYMT1, DEPDC1, KIF4A and CA4. Aberrations in cell cycle regulation facilitate sustained proliferative signalling and evasion of the growth suppressor, which are complementary hallmarks of cancers (Hanahan, 2022). The top 200 linear model genes were screened against the known cancer driver genes in Cancer Gene Census, yielding four hits: BUB1B, EBF1, PPARG, and RECQL4. RECQL4 is a key DNA helicase, with a vital role in the maintenance of genomic stability (Croteau et al., 2012). It has been found to be mutated and often upregulated in breast cancer (Luong et al., 2022), and its tumor-promoting activity has been observed in sporadic breast cancers with aggressive tumor behavior (Arora et al., 2016). Searching the top 200 MEGs against the Cancer Gene Census yielded two other hits: EGFR and QKI. EGFR is the first antitumor target to be identified, and known to be overexpressed in most of the TNBC and inflammatory breast cancers (Masuda et al., 2012), but associated with paradoxical function in metastatic cancer progression (Ali and Wendt, 2017). Significant downregulation of QKI has been noted in breast cancer relative to normal tissues, along with poor prognosis, which suggest its tumor-suppressor role (Cao et al., 2021). Expression of SLUG and QKI was correlated with epithelial to mesenchymal transition (EMT), and showed promise for use in breast cancer prognosis (Gu et al., 2019). Intersection of the top 200 linear model genes with the top 200 MEGs yielded 18 genes (including RECQL4), whereas intersection with the top 200 of the second linear model yielded 32 genes. We found 17 genes in common to all the three sets, including FAM13A, GABRD, and SORBS1. Supplementary File S15 presents the complete results. FAM13A is a hypoxia-induced gene in non-small lung cancer, increasing susceptibility to BC in a population-based cohort (Wei et al., 2019). Genes coexpressed with GABRD in colon cancer showed an enrichment for breast cancer and HPV infection pathway (Liu and Fang, 2021), hinting at a possible regulatory role for the monotonic expression of GABRD. Downregulation of SORBS1 in cancer samples was associated with increased metastasis and poor survival outcomes (Song et al., 2017). Stage-wise distribution of expression of representative consensus genes is presented in Supplementary File S16.

The 34 stage-salient candidate biomarkers identified here were cross-referenced with the Human Protein Atlas (Uhlen et al., 2017). We found 11 genes (2 stage-III salient genes and 9 stage-IV salient genes) annotated as ‘cancer related genes’, of which two stage-IV salient markers, namely EGR3 and KRT15, were specifically noted as prognostic markers of breast cancer (Supplementary File S17).

#### 4.1.2 Early-stage salient genes

Supplementary File S18 shows the expression distribution of early-stage salient genes in all the TCGA samples grouped by stage. Notice the curved trend in expression signifying salience of expression in an intermediate stage of cancer progression, not the terminal stage. Nicotine in tobacco exerts its action through nicotinic acetylcholine receptors, which initiate cell proliferation (Singh et al., 2011), according with the identification of CHRNA6 (neuronal nicotinic acetylcholine receptor) as stage-I salient here. The downregulation of CHRNA6 with cancer progression is supported by studies on nicotinic expression in non-small cell lung cancer progression, where expression of CHRNA6 was found higher in non-smokers than smokers (Lam et al., 2007). MMP10 is a member of the peptidase M10 family of matrix metalloproteinases, and could set the stage for cancer progression by facilitating tumor cell dissociation, augmenting migration/invasion capability, promoting endothelial cell tube formation, and inducing the expression of key angiogenic and metastatic factors (Zhang et al., 2014). Recently, Piskor et al. proposed that MMP10 in combination with MMP3 and CA-15 could be used as a biomarker panel for early-stage BC through a non-invasive approach (Piskór et al., 2020). Both these results accord with maximum expression of MMP10 in the early stages of cancer, reaffirming the effectiveness of our study design in identifying stage-salient markers. DEPDC1 is a novel cell cycle gene regulating apoptosis (Mi et al., 2015), whose over-expression signifies cancer progression in BC and its subtypes (Zhao et al., 2019; L et al., 2019). Here we have pinpointed the stage-II salience of DEPDC1 over-expression. COX7A1 is involved in mitochondrial metabolism and was identified as a tumor suppressor in invasive breast carcinoma, due to aberrant promoter hypermethylation (He et al., 2019). The stage-II salience of COX7A1 obtained in our studies supports its further exploration as a new biomarker and therapeutic target.

#### 4.1.3 Stage-III salient genes

Supplementary File S18 includes the expression distribution of stage-III salient genes in all the TCGA samples grouped by stage. It is known that KCNK15 is overexpressed in BC (S et al., 2013), specifically in Luminal A subtype, but downregulated in TNBC subtype (Dookeran et al., 2017). MFSD4 (major facilitator superfamily domain containing 4) has been identified as a tumor suppressor of cell motility and invasiveness (by influencing promoter methylation) and a biomarker of hepatic metastasis in gastric cancer (Kanda et al., 2016), correctly identified here as downregulated. CDH19 encodes a cell-cell adhesion receptor cadherin, essential to maintenance of intercellular connections, whose loss of function was observed in BC samples (Tervasmäki et al., 2014). Aligning with this result, CDH19 is seen here to be downregulated. CXCL5, a chemokine, was found to regulate bone colonization in metastatic BC via its functional target CXCR2 (R et al., 2019), and its downregulation here might need further review. Oncogenic expression of AKR7A3 in the late stages of BC is detrimental to the period of disease-free survival, and it is interesting to note its stage-III salient upregulation here (V et al., 2014). DEGS2 (delta (4)-desaturase sphingolipid 2) exhibits oncogenic expression in response to increased levels of ceramide in BC (Makoukji et al., 2015), which resonates with the findings here. Growth differentiation factor-5 (GDF5) regulates TGFβ-mediated pro-angiogenic signaling (Margheri et al., 2012), and its significant downregulation in the late stages here might set the stage for metastatic cancer. Oncogenic expression of FOXA1 (Forkhead box A1) enables widespread epigenetic reprogramming in ER metastatic BC (Fu et al., 2019), concordant with its overexpression here. Oncogenic expression of CST2 has been documented to promote bone metastasis in breast cancer (Blanco et al., 2012), borne out by its upregulated stage-III salience here.

#### 4.1.4 Stage-IV salient genes

Supplementary File S18 includes the expression distribution of stage-IV salient genes in all the TCGA samples grouped by stage. A monotonic trend of downregulation culminating in a stage-IV extremum is discernible. Suzuki et al. examined the role of EGR3 in BC and concluded that its overexpression in concert with the expression of other genes is necessary to establish invasive and metastatic BC (Suzuki et al., 2007), which is in contradiction to the consistent downregulation seen here. FOS and FOSB showed near-monotonic downregulation in mean expression here, which might require further examination in the context of BC subtypes (Lu et al., 2005; Bamberger et al., 1999). DUSP1 (dual specificity phosphatase 1 or MAPK phosphatase 1) is a tumor-suppressor in the MAPK pathway that mediates the dephosphorylation of ERK1/2 (Chen et al., 2011), and its downregulation seen here is likely to underpin sustained proliferative signalling. FREM1 has been identified as a tumor-suppressor, whose downregulation enabled metabolic shift and tumor infiltration (Li et al., 2020), a finding underlined by the monotonic downregulation seen here. HFM1, helicase for meiosis 1, was reported to be altered in tumors relative to control samples (Taylor et al., 2008), and seen to be a tumor-suppressor here. ABCA10 is a member of the active transmembrane transport family, and was recently implicated in the progression-free survival of epithelial ovarian sarcoma (Seborova et al., 2019), and appears to portray a tumor-suppressor role in the context of our findings. KLK5, a serine protease, is a known tumor-suppressor whose activation is a promising anticancer therapy via repression of the mevalonate pathway (Pampalakis et al., 2014). The downregulation of KCNA1 (a voltage-gated potassium channel subfamily member) has been correlated with breast cancer aggressiveness (Lallet-Daher et al., 2013), lending its stage-IV salience in our analysis. KRT15 is known as cytokeratin and has recently been shown to be closely associated with tumorigenesis. Overexpression of KRT15 (cytokeratin) was seen in colorectal and squamous cell skin cancers, but its low expression in BC (as seen here) has been significantly associated with poor prognosis (Zhong et al., 2021). The remaining stage-IV salient genes were found to be involved in tumor progression via processes such as including inflammation, angiogenesis, and EMT transition.

### 4.2 Improving histological subtyping

The distinction between IDC and ILC has previously frustrated learning algorithms. An XGBoost model with 147 clinical, histopathological, mammogram features, and sonographic features has been reported with an internal testset accuracy of 0.84 on the binary classification problem (Vy et al., 2022). An AutoML deep-learning approach for identifying IDC samples alone from whole slide images yielded 0.85 accuracy on an independent dataset (Zeng and Zhang, 2020). Another study for classifying IDCs as early-stage vs. late-stage yielded an AUROC of 0.47 on the external validation (Roy et al., 2020). In this context, the external validation of our model yields a significant improvement on the state-of-the-art. However the limited sensitivity to ILC samples (conversely, specificity to IDC samples) in the external dataset presents an outstanding challenge in the histological classification of breast cancer from molecular information. Some noteworthy features from this model include: (i) CDH1 (E-cadherin), whose germline mutations were strongly associated with lobular carcinoma (Corso et al., 2018), was found to have a specific downregulated expression signature in ILC samples; (ii) CCL14, which is known to promote angiogenesis and metastasis in breast cancer (Li et al., 2011), was found oncogenic in expression across both histological subtypes. Further improvements to histological subtyping models could come from:

- stacking the classifiers: the ensemble of XGBoost and neural network used herein showed that the classifiers disagree on many instances preventing a consensus classification. In such cases, improvements to the performance tradeoff could be achieved by ‘weighting’ the contribution of the two constituent models to the final prediction.
- using cross-modal features, including from early integration of multi-omics and spatial dynamics at cellular resolution.

### 4.3 Commercial gene panels for breast cancer

Available genomic assays (commercial or otherwise) for prognosticating breast-cancer adjuvant chemotherapy include the following gene-signature panels:

1. Prosigna (50 genes from PAM50 for intrinsic subtype classification, 8 housekeeping genes used for signal normalisation, 6 positive controls, and 8 negative controls)
2. OncotypeDX (16 cancer related +5 reference gene panel),
3. EndoPredict (3 proliferation-associated genes, 5 hormone receptor-associated genes, 3 reference genes),
4. MammaPrint (70 cancer-related genes; prognostic only) (van de Vijver et al., 2002),
5. Breast Cancer Index (exploring benefit of extension of adjuvant hormonal therapy beyond 5 years based on a 11-gene signature),
6. HER2DX (exploring benefit of neoadjuvant systemic therapy in HER2+ BC based on a 4-gene signature) (Prat et al., 2022), and
7. Guardant 360 (Guardant, 2020) and Foundation One Test (Foundation Medicine, 2020) (using liquid biopsies of circulating cell-free tumor DNA to profile 70+ biomarkers at progression).

Scanning the signatures in these genomic assays against the ten features used in our ‘normal’ vs. ‘cancer’ model yielded: two genes in common with Prosigna (FOXA1, MMP11), one gene with OncotypeDX (MMP11), one gene with HER2DX (NEK2), and one gene with Breast Cancer Index (NEK2). Scanning these signatures against the 16 features used in our molecular subtyping model yielded: four genes with Prosigna (ERBB2, FOXA1, GRB7, MLPH), four genes with HER2DX (ERBB2, GRB7, STARD3, AGR3), two with OncotypeDX (GRB7 and ERBB2), and two with Guardant360 (ERBB2, GATA3). Scanning these signatures against the 24 features used for histological subtyping yielded: one gene with Guardant360 (CDH1). Scanning these signatures against the five features used in the non-metastatic vs. metastatic model did not identify anything in common. These results indicate that the models developed in this work are novel and deserving of clinical validation. A summary of the existing gene-signature diagnostic tests (with their indications and outcomes) together with a comprehensive comparative study is provided in Supplementary file S19.

### 4.4 BC-predict

To transition the results obtained from our studies, we developed BC-Predict which serves the models developed in a cascade inference engine and provides a comprehensive characterization of the given sample (Figure 2). The BC-predict web-server is built on Rshiny (Beeley, 2016) and deployed for academic research at https://apalania.shinyapps.io/BC-Predict. All predictions are accompanied by prediction probabilities to provide confidence for the predicted class. Documentation and video tutorial for the use of BC-Predict are also provided. BC-Predict generates a unified readout that could nominally support medical decision-making contingent to clinical validation and further refinement. An alternative modeling process that used a nested stratification structure instead of sequential stratification was also investigated but did not yield an improvement. Though the cancer vs. normal model improves on the benchmark, iterative refinement and better datasets could yield further performance improvements for all models. Below we present a systematic enumeration of the limitations of our models and suggested coping strategies:

1. The metastatic model does not distinguish among the stages in pre-metastatic cancer. A refinement may be necessary to discriminate between the early-stage cancers (stages I and II) and stage-III cancers among the pre-metastatic cancers.
2. The molecular subtype model lumps ‘Luminal A’ and ‘Luminal B’ into the ‘Luminal’ class. Both luminal A and B are HER2-and ER+, however the A subtype is PR+ and the most common molecular subtype comprising 50%–60% of breast cancers whereas the B subtype accounts for 15%–20%, mostly PR- and with low levels of Ki-67. Thus Luminal B has distinctly better prognosis than Luminal A. Increased data size and quality could afford production of better models that differentiate between these subtypes.
3. The ILC histological subtype tends to be radiologically and clinically hard to detect, manifesting more as thickening with occult mammogram rather than mass, hence research is urgent to improve the detection of this class, as discussed above.
4. The identified gene-signature panels could be enhanced with the inclusion of reference gene normalization, for more robust predictions.
5. In addition, all models would need to be fine-tuned for distribution shifts possible in different populations, though the identity of the biomarkers is likely invariant. Initiatives akin to the Indian Cancer Genome Association (Dixit and Sadanandam, 2021) could facilitate model monitoring and adaptation.

Gene-signature methods remain the clinical standard for both their effectiveness and utility, and works such as ours are a step forward in resolving difficult challenges. Such diagnostic models need to be clinically validated and approved for use by national regulatory bodies such as the FDA (Food and Drug Administration, USA), MHRA (Medicines and Healthcare products Regulatory Agency, UK), EU MDR (European Union Medical Device Regulation), NMPA (National Medical Products Administration, China) and CDSCO (Central Drugs Standard Control Organization, India). Models are complicated by cohort selection bias; for e. g., breast cancer in Black population presents in younger patients and more difficult to treat forms (aggressive, grade-III, TNBC or HER2+) than in Hispanic population, with poorer prognosis. Also, metastatic breast cancer is rarely synchronous (more metachronous) in developed nations as opposed to metastatic cancer on presentation in emerging nations. In addition to these variations, AI-based diagnostic modalities need to contend for the interplay of risk factors that could enable or confound the predictions: pre-menopausal vs. post-menopausal, node-positive or not, complete hormonal profile and NPI score. Clinical validation of BC-Predict would involve the synthesis and use of specific forward and reverse primers for each model feature to perform qRT-PCR on the isolated RNA of resected biopsy sample from a patient. Post-quantification (normalized counts) and log_2_ transformation, the inference model may be served to yield a prediction. Prior to such deployment, calibration of qRT-PCR may be necessary and could involve reference genes as used in, say, NOVAprep-miR-Cervix (Kniazeva et al., 2023).

In summary, we have developed performant *de novo* models to characterise breast cancer heterogeneity agnostic of hypothesis. The candidate stage-salient biomarkers could play a role in the progression of breast cancer, whose varying manifestations underlie differential response to treatment regimens. Developing models from minimal feature spaces has several advantages, chief among them being sensitivity to heterogeneous individual presentation, and generalization to out-of-domain population. One example of this in the present study is the performant external validation of the Molecular Subtype model on the TNBC-only African-enriched multiethnic international cohort (25/26 samples correctly identified). It is noteworthy that TNBC is also the most common molecular subtype in the Indian subcontinent, and has frustrated drug discovery programs with few druggable targets. It may be noted that the use of mere five features in the metastatic model mitigates against the limited datasets available, and offers realistic prospects for useful generalization in clinical diagnostics. Validation analysis with miRNA strongly supported DEPDC1, FOSB and DUSP1 as potential biomarkers for metastasis. More generally, the candidate model features identified here could provide novel hypotheses for chemotherapy and immunotherapy investigations. We would like to acknowledge that the late-integration of multi-omics has not consistently provided conclusive evidence for the features used in the models, yielding possible directions for future investigations. Our study overcomes certain limitations of earlier models, namely reporting of balanced performance metrics, availability for academic research, and inclusion of external validation. The confidence returned by BC-Predict predictions could be used to safeguard against weak and uncertain evidence, addressing the hazard with AI/ML modelling (Yao et al., 2022). The clinical translation of AI/ML models would be a step forward for personalized medicine, necessitating adequate regulation to ensure the benefits of AI for all (El Naqa et al., 2023; Hickman et al., 2021). Validation and assurance of model quality could alleviate the risks of distribution drift and cohort selection bias, and pave the way for clinically effective decision support aids in precision oncology centers. The realisation of software-as-medical-devices promises to revolutionize the diagnosis, triage, and treatment of cancers.

## 5 Conclusion

Assessment of low-risk genetic factors unmasks induced vulnerabilities, and early-stage characterization of breast cancer heterogeneity constitutes the premise for personalized and targeted precision medicine. In this work, we have developed *de novo* models for addressing key problems in breast cancer heterogeneity based on public-domain expression datasets. Using custom protocols to identify features of interest to each problem, we have trained, optimised and externally validated the models. Our analysis has yielded novel and stage-salient drivers of cancer progression, including two stage-I salient genes (CHRNA6, MMP10), two stage-II salient genes (DEPDC1, COXA1), ten stage-III salient genes (including AKR7A3, FOXA1, CXCL5 and GDF5) and 20 stage-IV salient genes (including FREM1 and HFM1). We have developed solutions to four problems of interest in characterizing breast cancer heterogeneity: (i) ‘cancer’ vs. ‘normal’ based on 10 features (2 stage salient genes and 8 top linear model genes) with balanced accuracy ∼97.42% on external validation; (ii) non-metastatic vs. metastatic based on 5 features with balanced accuracy ∼88.22% on external validation; (iii) molecular subtyping (namely Luminal, HER2+, and TNBC) based on 16 features with balanced accuracy ∼88.79% on external validation; and (iv) histological subtyping (IDC vs. ILC) based on 24 features with ensemble accuracy ∼94.23% on external validation. We have validated our results in multiple modalities. Based on these outcomes, we have developed an inference engine BC-Predict, which serves the best models developed for each problem, upon an input instance of expression data from a patient sample. BC-Predict is available for academic and non-commercial purposes as an experimental predictive aid for characterization of breast cancer heterogeneity based on minimal expression information, and subject to refinement with new knowledge. In conclusion, we have identified various novel candidate biomarkers of heterogeneous breast cancers that have been embedded into one integrated and validated cascade model that could pave the path to expediting personalized differential diagnosis and early-stage cure.

## Data Availability

The data presented in the study are deposited in the figshare repository, accession number https://doi.org/10.6084/m9.figshare.25282906.v2
